# A comparative genomics study of genetic products potentially encoding ladderane lipid biosynthesis

**DOI:** 10.1186/1745-6150-4-8

**Published:** 2009-02-16

**Authors:** Jayne E Rattray, Marc Strous, Huub JM Op den Camp, Stefan Schouten, Mike SM Jetten, Jaap S Sinninghe Damsté

**Affiliations:** 1 NIOZ Royal Netherlands Institute for Sea Research, Department of Marine Organic Biogeochemistry, P.O. Box 59, 1790 AB Den Burg, Texel, The Netherlands; 2 Department of Microbiology, IWWR, Radboud University Nijmegen, Toernooiveld 1, 6525 ED Nijmegen, The Netherlands

## Abstract

**Background:**

The fatty acids of anaerobic ammonium oxidizing (anammox) bacteria contain linearly concatenated cyclobutane moieties, so far unique to biology. These moieties are under high ring strain and are synthesised by a presently unknown biosynthetic pathway.

**Results:**

Gene clusters encoding enzymes of fatty acid biosynthesis in the anammox bacterium *Kuenenia stuttgartiensis *and 137 other organisms were analysed and compared *in silico *to gain further insight into the pathway of (ladderane) fatty acid biosynthesis. In *K. stuttgartiensis *four large gene clusters encode fatty acid biosynthesis. Next to the regular enzyme complex needed for fatty acid biosynthesis (FASII), the presence of four putative S-adenosyl-methionine (SAM) radical enzymes, two enzymes similar to phytoene desaturases and many divergent paralogues of *β*-ketoacyl-ACP synthase (*fabF*) were unusual. Surprisingly, extensive synteny was observed with FASII gene clusters in the deltaproteobacterium *Desulfotalea psychrophila*. No ladderane lipids were detected in lipid extracts of this organism but we did find unusual polyunsaturated hydrocarbons (PUHC), not detected in *K. stuttgartiensis*.

**Conclusion:**

We suggest that the unusual gene clusters of *K. stuttgartiensis *and *D. psychrophila *encode a novel pathway for anaerobic PUFA biosynthesis and that *K. stuttgartiensis *further processes PUFA into ladderane lipids, in similar fashion to the previously proposed route of ladderane lipid biosynthesis. However, the presence of divergent paralogues of *fabF *with radically different active site topologies may suggest an alternative pathway where ladderane moieties are synthesised externally and are recruited into the pathway of fatty acid biosynthesis.

**Reviewers:**

This article was reviewed by Dr Michael Galperin (nominated by Prof E. Koonin), Dr Andrei Osterman and Dr Jeremy Selengut.

## Background

**Figure 1:**
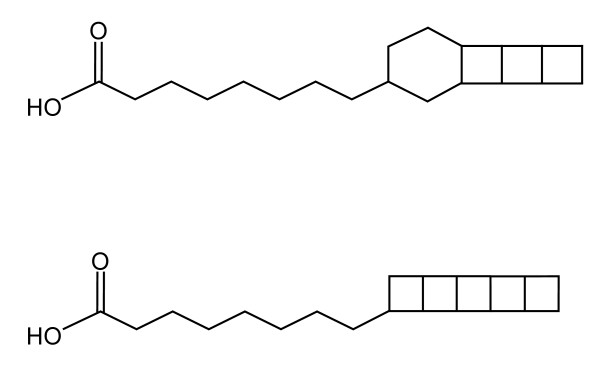
**C_20 _ladderane fatty acids containing 3 or 5 linearly concatenated cyclobutane moieties**.

Anammox (**an**aerobic **amm**onium **ox**idizing) bacteria are unusual specimens of the phylum *Planctomycetales *[1]. They play an important role in the oceanic nitrogen cycle [2-5] and are applied at industrial scale to remove ammonium from wastewater [6,7]. Anammox catabolism uses 1 mole of ammonia and 1.32 moles of nitrite to produce dinitrogen gas in the absence of oxygen [8]. Exploitation of this energy source under anaerobic conditions is thought to have resulted in the evolution of unique cellular architecture [9,10]. For example, the cell membranes of anammox bacteria are comprised of linearly concatenated cyclobutane moieties, aptly named 'ladderane' lipids [11]. Ladderane lipids consist of units containing either 3 or 5 linearly fused cyclobutane moieties that can be synthesised into a variety of lipid structures; fatty acids (Figure 1), alcohols, mono-ethers, di-ethers and mixed ether-ester phospholipids [12,13].

The first hypothesis for the mechanism of ladderane lipid biosynthesis was proposed by Sinninghe Damsté et al. and involved ring closure of a C_20 _polyunsaturated fatty acid at C_9 _and C_20 _(C_12 _macrocycle formation) with subsequent carbon-carbon bonding, to create the linearly fused cyclobutane moiety [13]. Due to the structural similarity of the ladderane lipid moieties, the number of cyclization steps could then be increased or reduced to synthesise the 3 or 5 cyclobutane containing moieties. Mascitti and Corey [14] suggested that ladderane biosynthesis could occur via a cascade type polycyclization, using a substrate like the allenic C_20 _fatty acid 9,10,12,16,18,19-docosahexaenoic acid. Despite the fact that allenic fatty acids are rare in the natural environment and to the best of our knowledge have only been reported to occur higher plants [15-17], anaerobic bacteria including other *Planctomycetes *have been reported with the ability of synthesising lipids containing multiple double bonds [18-20].

**Figure 2:**
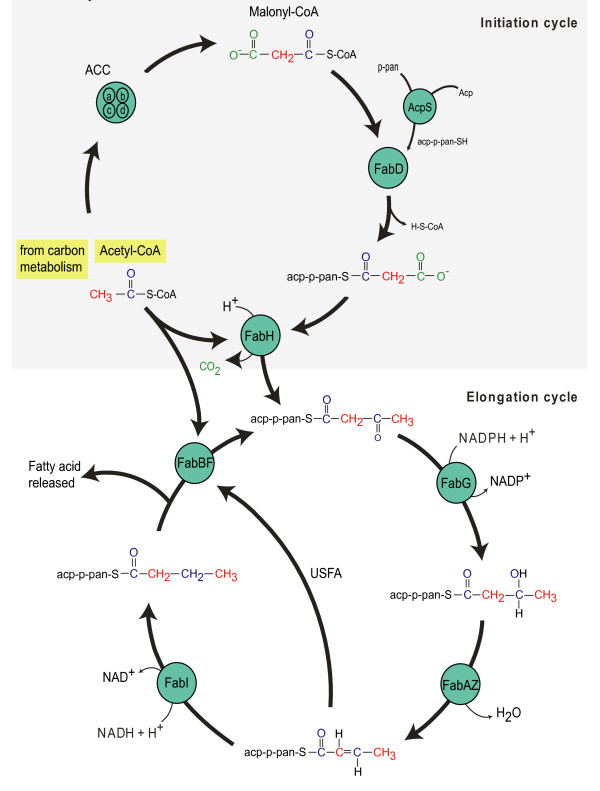
**Schematic diagram of the flow of carbon during type II fatty acid biosynthesis in *Escherichia Coli*, adapted from White et al. **[21]. Table 1 contains enzyme name abbreviations. USFA, pathway of unsaturated fatty acid biosynthesis bypasses enzyme FabI.

Fatty acid synthesis in bacteria and plants usually occurs via type II fatty acid synthesis (FASII), as recently reviewed by White et al. [21]. In *Escherichia coli*, FASII is made up of seven separate soluble proteins, each encoded by a discrete gene. Figure 2 displays the sequence of events during FASII biosynthesis, with additional information given in Table 1. A crucial component in this pathway is the acyl carrier protein (ACP) that transports lipid intermediates (as ACP thioesters) between different enzymes in the FASII pathway. In the initiation module, ACP is activated, condensed with malonyl CoA (using Malonyl-CoA:ACP transacylase, FabD) and undergoes the first condensation reaction via *β*-Ketoacyl-ACP Synthase III (FabH). After leaving the initiation cycle the acetoacetyl-ACP intermediate enters the elongation cycle (via FabB or FabF) where four enzymatic reactions catalyse the growth of the lipid chain by two carbons per pathway cycle. The gene products of the paralogues *fabB *and *fabF*, are similar in both protein crystal structure and catalytic function. These two isozymes condense the growing acyl-ACP with malonyl-ACP to extend the chain by two carbon atoms, and can differentiate between the entrance of a growing lipid chain or a new lipid chain. However, it has been demonstrated that mutants of *E. coli *lacking in FabB are incapable of unsaturated fatty acid synthesis [22] and mutants lacking in FabF were unable to control fatty acid synthesis in response to temperature [23].

**Table 1:** Gene products and proteins encoding fatty acid synthesis in *E. coli *[21].

Protein	Full name protein	Gene Product	*K. stuttgartiensis *Locus Tag	Number of paralogues
ACP	Acyl carrier protein	*acpP*	*Kustd1387*	*2*
AacpS	ACP synthase	*acpS*	*Kuste2802*	*0*
AccA	Acetyl-CoA carboxylase (ACC)	*accA*	*kustd1493*	*0*
AccB	Acetyl-CoA carboxylase (ACC)	*accB*	*kuste2852*	*0*
AccC	Acetyl-CoA carboxylase (ACC)	*accC*	*kuste2853*	*0*
AccD	Acetyl-CoA carboxylase (ACC)	*accD*	*kustd1647*	*0*
FabD	Malonyl-CoA ACP transacylase	*fabD*	*kustd1388*	*0*
FabH	*β*-Ketoacyl-ACP synthase III	*fabH*	*kustd1389*	*0*
FabB	*β*-Ketoacyl-ACP synthase I	*fabB*	*kuste2805*	*0*
FabF	*β*-Ketoacyl-ACP synthase II	*fabF*	*kustd1386*	*7*
FabG	*β*-Ketoacyl-ACP reductase	*fabG*	*kuste3341*	*6*
FabA	*β*-Hydroxydecanoyl-ACP dehydratase	*fabA*	*none*	*n.a.*
FabZ	*β*-Hydroxyacyl-ACP dehydratase	*fabZ*	*kuste3604*	*2*
FabI	Enoyl-ACP reductase I	*fabI*	*Kustd2023*	*0*
FabK	Enoyl-ACP reductase II	*fabK*	*none*	*n.a.*
FabR/FadR	Transcriptional activator/repressor	*fabR/fadR*	*none*	*n.a.*

Also listed are the equivalent gene sequences and number of paralgoues found in *K. stuttgartiensis*.

After FabB and FabF, the lipid intermediate undergoes reduction of the carbonyl group by the NADPH dependant *β*-ketoacyl-ACP reductase (FabG), producing the intermediate *β*-ketoacyl-ACP. The isozymes FabA and FabZ (*β*-hydroxyacyl-ACP dehydratases) perform the third step in the elongation cycle, the initiation of unsaturated fatty acid biosynthesis in the growing lipid chain. *β*-hydroxyacyl-ACP is initially dehydrated to yield a double bond in the C_2_–C_3 _region, creating *trans*-2-butenoyl-ACP. This product can then proceed in the cycle to have the double bond reduced by FabI (enoyl-ACP reductase), or alternatively, FabA can isomerise e.g. *trans*-2-decenoyl-ACP to *cis*-3-decenoyl-ACP that bypasses FabI and is used by FabB to initiate the first cycle of unsaturated fatty acid biosynthesis [24]. At the last step of the cycle, FabI reduces the double bond (C_2_–C_3_) in *trans*-2-decenoyl-ACP, using NADPH as the electron donor. The fatty acid then resides in the elongation cycle until the required acyl chain length is achieved, at which point the fatty acid is released from the cycle to undergo further processing into intact membrane lipids. FASII is also a versatile pathway; it can be used for synthesising various lengths of normal straight chain fatty acids, hydroxy, *iso *and *ante-iso *branched fatty acids and non-lipid cellular metabolites including molecules for quorum sensing and lipoic acid [21].

Initial analysis of the *K. stuttgartiensis *genome has provided several clues to the potential pathway of ladderane lipid biosynthesis [25]. In *K. stuttgartiensis*, four gene clusters putatively encode fatty acid biosynthesis. Apart from encoding typical FASII enzymes, three clusters additionally contain genes encoding unusual S-adenosyl-methionine (SAM) radical enzymes. The ability of these enzymes to catalyse diverse and unusual reactions [26-28] was the basis of the proposal that SAM radical enzymes perform a key role in the biosynthesis of ladderane lipids [25]. In addition to SAM radical enzymes, other genes not normally associated with fatty acid biosynthesis were also detected. In this study we aimed to provide further insight into ladderane biosynthesis in anammox bacteria, by applying detailed analysis and functional prediction of *K. stuttgartiensis *gene products and comparing these with fatty acid gene clusters from a diverse range of bacterial and archaeal genomes.

## Methods

### Database retrieval and data assessment

Genomic information from the *K. stuttgartiensis *genome was extracted from the Protein Extraction, Description and Analysis Tool PEDANT [29] and National Centre for Biotechnology Information (NCBI) databases [30]. Amino acid sequences encoding lipid biosynthesis in other organisms were retrieved from a selection of online databases; The Kyoto Encyclopaedia of Genes and Genomes (KEGG) [31], The Institute for Genomic Research (TIGR) [32] and NCBI [30]. Basic logical alignment search tool (BLAST) searches were performed using KEGG blast [31] and NCBI blast [33]. Amino acid sequences were analysed using MEGA 3.1 [34] and aligned using Clustal W [35] with a Gonnet protein weight matrix. Protein active sites were identified by comparison to predetermined protein crystal structures available on the Research Collaboratory for Structural Bioinformatics (RCSB) protein data bank (PDB) [36]. For phylogenetic analysis, amino acid sequences were aligned using Clustal W and manually refined. Minimum evolution phylogeny with bootstrapping (500 re-samplings) was performed using MEGA 3.1 [34]. Protein active sites were determined using (Conserved Domain Architecture Retrieval Tool (CDART) [37] and the conserved domain database (CDD) [38]. Alignment of sequences using this database, are related to the percentage similarity of residue conservation patterns from other members of that particular protein family. Protein sequences were also blasted using Conserved Domain Search (CD-Search) where the proteins are compared using similarities in architecture rather than sequence alignment [39].

### Comparative genomics

Lipid gene clusters from the genomes of a representative selection of 137 different organisms publicly available in the databases in June 2006 were compared with the four gene clusters of *K. stuttgartiensis*. The genomes were screened for gene clusters that contained at least two canonical fatty acid biosynthesis genes and two of the accessory genes present in the gene clusters of *K. stuttgartiensis*. Genes were considered homologous when they shared at least 20% amino acid identity over at least 70% of the length [25]. The screening was performed with a custom script that used, as the input, reciprocal BLAST searches of the *K. stuttgartiensis *genes against the 137 genomes.

### Biomass culturing and extraction and analysis of lipids

Biomass of the anaerobic psychrophilic sulphate-reducing bacteria bacteria *Desulfotalea psychrophila *was cultured at DSMZ (Deutsche Sammlung von Mikroorganismen und Zellkulturen GmbH), and grown according to DSMZ protocols [40]. Biomass was frozen, freeze-dried, ultrasonically extracted, saponified and analysed for lipids as described previously [13]. The total lipid extract was analysed using gas chromatography (GC) and gas chromatography mass spectrometry (GC/MS) as described previously [41]. For identification of molecular ions of selected compounds the lipid extract was analysed by GC/Chemical Ionization (CI)/MS. These were performed using a HP 6890 Series GC System (Hewlett Packard) equipped with a 25 m × 0.32 mm × 0.12 μm CPSil 5CB (Chrompack) silica column coupled to an HP 5973 Mass Selective Detector. Helium was used as a carrier gas and CH_4 _as the reaction gas.

Ladderane lipid analysis was performed using the high performance liquid chromatography (HPLC)-MS/MS procedure of Hopmans et al. [42]. Stable carbon isotope analysis of individual lipids was performed as described in detail elsewhere [43]. The isotopic composition of the extra carbon added during sample derivatization was determined using derivatizing agents with known isotopic compositions and subsequently adjusting the isotopic compositions of the fatty acids and alcohols. δ^13^C values of the sodium lactate crystals used as the carbon source for *D. psychrophila *were determined using elemental analysis isotope ratio monitoring (EA-IRM)/MS as described previously [41].

## Results

### Comparative genomics analysis

*K. stuttgartiensis *contained equivalents of most genes that encode FASII biosynthesis in *E. coli *(Table 1; Figure 2). However, genes encoding FabA were not located, suggesting that *K. stuttgartiensis *is incapable of producing unsaturated fatty acids via the conventional FASII pathway. Most of the genes that were present existed as a single copy, but multiple paralogues of acyl carrier protein (*acp*, 3 copies) and *β*-ketoacyl-ACP synthase (*fabB *and *fabF*, 7 copies) were identified in the lipid gene clusters. Multiple copies of *acp *were also present in *Mycobacteria *and in organisms employing polyketide biosynthesis (e.g. *Shewanella *sp.). A distant relative to *K. stuttgartiensis*, the planctomycete *Rhodopirellula baltica *and the unrelated sulphate-reducing deltaproteobacterium *Desulfotalea psychrophila *both contained 5 *fabB/F *paralogues.

**Figure 3:**
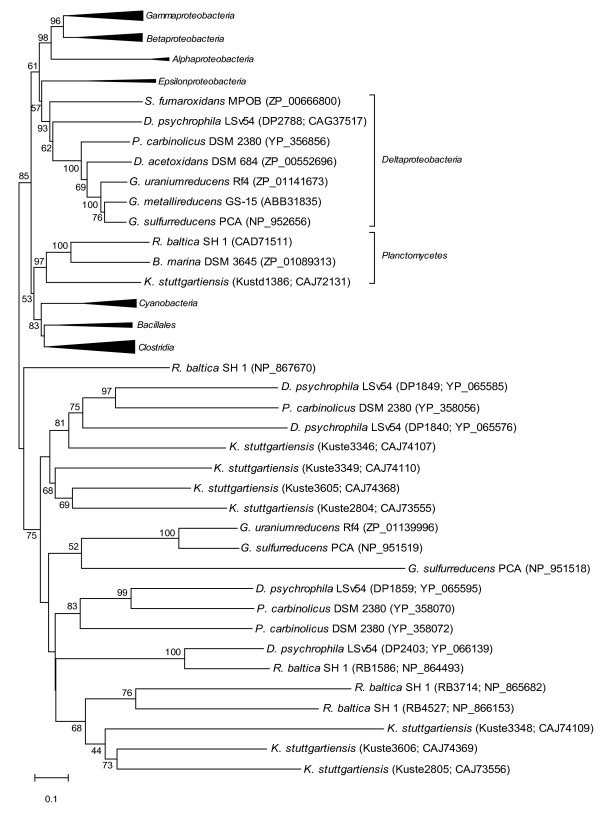
**A minimum evolution phylogenetic tree of *fabB *and *fabF *displaying distribution of the protein paralogues**. Bootstrap values correspond to the consensus of 500 replications, final tree was based on bootstrap consensus. Missing gaps were treated using pairwise deletion. Accession numbers of all proteins and locus tags of gene products mentioned in text are shown.

We studied in detail the active site residues essential for the functioning of *β*-ketoacyl-ACP synthases, since deviation in active site residues could point to the use of alternative substrates by different paralogues. The *K. stuttgartiensis *paralogues Kuste3606, Kuste2805 and Kuste3348 display significant differences in the 'active site' residues (Table 2), whereas the primary structures of the active site of the other five *fabF *sequences were highly conserved. As seen in Table 2, the *R. baltica fabF *paralogues RB 3714 (NP_865682) and RB 4527 (NP_866153) also miss the active site cysteine (Cys163) that is substituted for a negative hydrophilic amino acid, Asp and Glu in the respective samples. Deviations in the sequences of these two paralogues of *R. baltica *are very similar to those observed in *K. stuttgartiensis *and is consistent with the grouping of these paralogues together in the phylogenetic tree in Figure 3. The upper section of the minimum evolution phylogenetic tree shows the 'standard' *fabF *gene sequences from a selection of bacteria (Kustd1386 in *K. stuttgartiensis*), and the middle section shows the grouping of unusual *fabF *paralogues that have conserved active sites. The lower section of the tree displays the putative *fabB *(Kuste2805) and *fabF *paralogues (Kuste3348, Kuste3606) all of which are classified by NCBI CDD [38] as having similarities to beta-ketoacyl protein synthase, but do not have the same essential active sites found in the *E. coli fabB/F *crystal structure. Therefore, information in Table 2 and Figure 3 indicates that Kuste3348, Kuste3606 and Kuste2805 are unlike the other *fabB *or *fabF *paralogues in *K. stuttgartiensis *or other organisms analysed, thus suggesting a different enzyme substrate and function. The remaining part of the recognised FASII gene products were found to be usual with respect to amino acid sequence, including conserved residues and the number of paralogues.

**Table 2:** Deviations in enzyme active sites in *fabB *and *fabF *paralogues in *K. stuttgartiensis*, *R. baltica*, *G. sulfurreducens *in comparison to 'typical' *fabB/F *sequences in e.g. Kustd1386 (CAJ72131) or the structurally determined *EcfabB *(YP_540234).

**Gene sequence**	**Active site amino acids from *fabB/F *sequence**							
Typical *fabB/F *sequence and function	Active site	Decarb-oxylation	Unknown	Unknown	Electronic charge on histidine ring	Decarb-oxylation	Electronic charge on histidine ring	Guides acyl chain entry into binding pocket
	
	*Cys163*s	His298*	*Thr300*	*Thr302*	Lys328*	His333*	*Glu342*‡	**Phe392**a

Kuste3606 *CAJ74369*	**Val163**	*Asn298*‡	*Tyr300*a	**Gly302**	Lys328*	*Glu333*‡	*Gln342*‡	Arg392*

Kuste2805 *CAJ73556*	**Phe163**a	*Asn298*‡	*Asn300*‡	*Cys302*s	Lys328*	*Glu333*‡	*Gln342*‡	**Phe392**a

Kuste3348 *CAJ4109*	*Ser163*	*Ser298*	**Pro300**i	**Phe302**a	*Asp328*‡	*Asn333*‡	*Asn342*‡	*Ser392*

*R. baltica* NP 865682	*Asp163*‡	His298*	**Met300**s	*Asp302*‡	**Ile328**	His333*	**Gly342**	*Ser392*

*R. baltica* NP 866153	*Glu163*‡	His298*	**Leu300**	*Asp302*‡	Lys328*	*Asn333*‡	*Glu342*‡	**Pro392**i

*G. sulfurreducens* NP 951518	**Phe163**a	**Pro298**i	**Ala300**	**Ala302**	**Gly328**	Arg333*	**Ala342**	-

Functional role of amino acids taken from White et al. [21]. Key; *Italics = hydrophilic (polar amino acids)*, Bold = hydrophobic (non-polar amino acids), (*) = Basic, (‡) = Acidic or amide, (s) = Sulphur containing, (a) = Aromatic, (i) = Imino acid.

**Figure 4:**
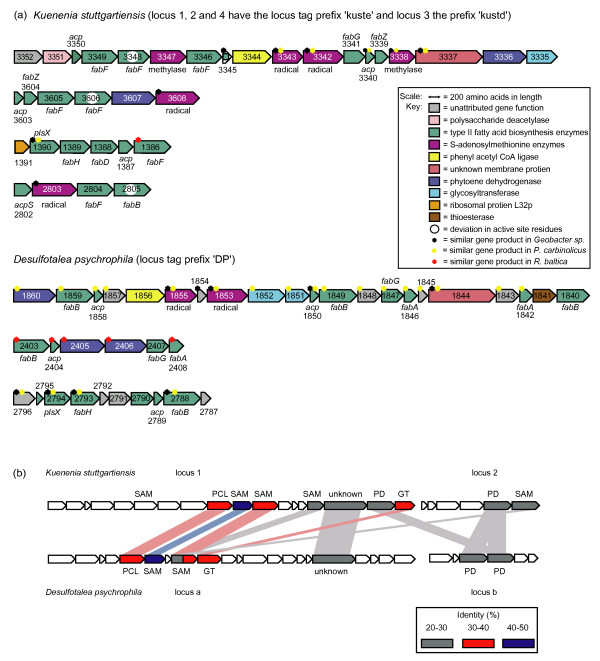
**(a) Functional assignment of proteins in loci potentially encoding fatty acid biosynthesis in *K. stuttgartiensis *and *D. psychrophila***. In both organisms FabI was located in unrelated gene clusters that have not been displayed below. (b) Pairwise comparison (identities) of unusual proteins found in locus 1 and 2, and loci a and b, in panel 4a. Pairwise comparisons were conducted using BLAST with a BLOSUM62 matrix file and gap penalties of 11/1 (existence/extension). SAM, S-adenosyl-methionine; PCL, phenylacetyl CoA ligase; OX, oxidoreductase; GT, glycosyltransferase; PD, phytoene dehydrogenase.

*K. stuttgartiensis *contained FASII gene clusters with six genes encoding putative SAM radical or methylase enzymes and two genes with some homology to phytoene dehydrogenases. To determine just how unusual this combination is, we searched databases for the occurrence of similar gene clusters in other organisms and found that such clusters were rare but not unique. Some deltaproteobacteria (*D. psychrophila*, members of the genus *Geobacter *and *Pelobacter carbinolicus*) contained similar gene clusters (Figure 4a). The synteny with *D. psychrophila *was striking, apart from the presence of similar SAM and 'phytoene dehydrogenase' genes, a gene encoding a new membrane protein with unknown function and a 'phenylacetyl-CoA ligase' were also in synteny, the pairwise identity of these 'unusual' enzymes is shown in Figure 4(b).

**Figure 5:**
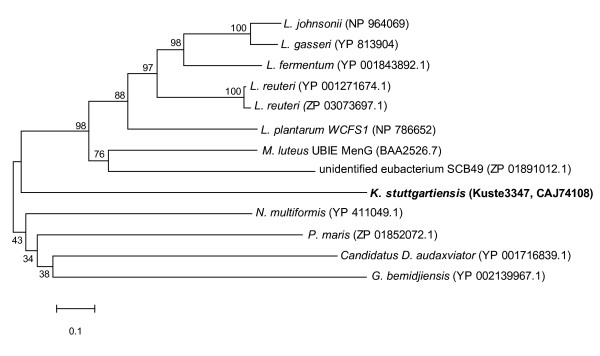
**A minimum evolution phylogenetic tree of closest relatives of Kuste3347, an unusual methyl transferase (obtained using closest matches in BLAST)**. Bootstrap values correspond to the consensus of 500 replications, final tree was based on bootstrap consensus. Missing gaps were treated using pairwise deletion. Accession numbers of all proteins and the locus tag of the *K. stuttgartiensis *gene product are shown. For comparative purposes the experimentally characterised UBIE MenG and methyl transferases with other putative functions are shown.

A small number of open reading frames in the *K. stuttgartiensis *gene clusters were not in synteny with gene clusters in *D. psychrophila *and had little sequence similarity with open reading frames in other organisms. Proteins with no formally assigned function were Kuste3352 (an unknown protein containing a domain classified under the VacJ (lipoprotein like) superfamily), Kuste3351 (a conserved hypothetical protein with domains similar to a polysaccharide deacetylase), Kuste3347 (a hypothetical protein with conserved domains indicative of a SAM methyl transferase), Kuste2803 (a hypothetical protein with conserved domains indicative of a SAM radical enzyme with an additional B12 binding domain) (Figure 4a). In particular, Kuste3347 was unlike other currently defined methyl transferases. To further investigate, this we constructed a minimum evolution phylogenetic tree of closest relatives of Kuste3347 (Figure 5). Homology was previously established by using sequences with a statistically significant similarity and a consistent pattern of shared residues when multiply aligned. For comparative purposes the closest BLAST hits, including the experimentally characterised SAM methyl transferase MenG (UBIE MICLU) were used [44]. In Figure 5 the gene sequence of Kuste3347 appears to have evolved separately in comparison to the other methyl transferases involved in ubiquinone synthesis. The early divergence of Kuste3347 and large difference in sequence similarity suggests that this protein may have evolved a separate function, making it a candidate for the production of unusual lipids.

Apart from the FASII gene clusters, we also investigated if there were clusters encoding lipid synthesis via the polyketide or isoprenoid pathways. No gene products were located in the *K. stuttgartiensis *(or the *D. psychrophila*) genome that resembled polyketide synthases known to synthesise PUFA in marine organisms [45] or any other type of polyketide synthase-like enzymes. In *K. stuttgartiensis*, gene sequences encoding enzymes for isoprenoid synthesis were found for the 2-C-methyl-D-erythritol 4-phosphate (MEP or non-mevalonate) pathway and for the production of terpenoid lipids.

### Lipid analysis

The striking synteny of gene products encoding fatty acid biosynthesis in *K. stuttgartiensis *and *D. psychrophila *prompted us to investigate the lipid composition of *D. psychrophila*. Core lipid fractions of *D. psychrophila *were analysed for ladderane lipids using GC/MS and HPLC-MS/MS. Ladderane lipids were not identified in the *D. psychrophila *biomass. However, unusual polyunsaturated hydrocarbons (PUHC) C_31:8 _and C_31:9_, with molecular masses of 418 and 420 respectively, were identified using GC/MS with electron impact and chemical ionization. The PUHCs formed 5% of the total lipid fraction (Table 3). The naturally occurring ^13^C/^12^C isotope ratios of individual lipids have previously been used to discriminate between different pathways of lipid biosynthesis [46] and may indicate the pathway of PUHC biosynthesis. The δ^13^C values of the PUHCs and fatty acids were determined and their isotopic composition relative to the carbon source lactate was calculated. δ^13^C values of the PUHCs and the most abundant fatty acids are shown in Table 3. Similar fractionation (< 5‰) of the δ^13^C may indicate that lipids are biosynthesised via the same (or similar) pathways. Two of the isomers of C_16:1 _and C_18:1 _fatty acids have values in a similar range as the PUHC (-20.1 to -22.8‰) which, suggests that these lipids are synthesised via the same pathway, perhaps an altered version of the FASII synthesis. The wide range of depletions of saturated and unsaturated fatty acids (up to 20.5‰) indicates that they are synthesised from different precursors (i.e. different sources of acetyl CoA or a different monomer altogether), as no mechanism exists for the isotopic fractionation of double bonded carbons [47]. This experimental evidence indicates that monounsaturated fatty acids could be the precursors of the PUHCs in this organism, thereby supporting the conclusion from the *in silico *analysis that PUHCs could be synthesised in a novel anaerobic desaturase modification of the FASII pathway.

**Table 3:** Carbon fractionation of major lipids in *D. psychrophila *calculated relative to the carbon source, lactate (-28.9‰).

**Lipid/Substrate**	**% abundance in total lipid extract**	**Δ δ^13^C‰ relative to lactate**
C_31:8 _(PUHC)	4	-20.1
C_31:9 _(PUHC)	1	-20.4
C_14:0_	2	-8.0
C_16:0_	12	-11.0
C_16:1_	(39, 15^a^)	(-16.1, -20.8^a^)
C_17:1_	1	-13.5
C_18:1_	(2, 2^a^)	(-28.5, -22.8^a^)

PUHC; polyunsaturated hydrocarbon. Mean values of duplicate measurements of one culture are given; replicate values deviated by a maximum of ± 1 ‰.

^a ^two isomers, separated by retention time.

## Discussion

Ladderane lipids are a curiosity of biological chemistry. As far as we know they are biosynthesised only by anaerobic ammonium oxidizing (anammox) bacteria and their biosynthesis proceeds via an unknown biochemical pathway. Anammox bacteria are not available in pure culture and no genetic system is conceivable in the near future. Furthermore, the bacteria grow very slowly and experimental approaches are limited by a low supply of anammox cells. In the present study, ladderane biosynthesis was addressed via comparative analysis of metagenomic data of the anammox bacterium *K. stuttgartiensis *and stable isotope analysis.

Pathways for ladderane biosynthesis were previously proposed on theoretical grounds, involving ring closure of a C_20 _polyunsaturated fatty acid at C_9 _and C_20 _(C_12 _macrocycle formation) and subsequent carbon-carbon bonding [13] that could proceed via cascade type polycyclization, with a substrate like the allenic C_20 _fatty acid 9,10,12,16,18,19-docosahexaenoic acid [14]. Nouri and Tantillo have suggested a six-step polycyclization mechanism that could lead to ladderane formation [48].

From a genomic perspective, it was noted previously that the operons for fatty acid biosynthesis in the anammox bacterium *K. stuttgartiensis *were larger than found in other bacteria and encoded a number of SAM radical enzymes. These SAM radical enzymes might provide extra functionality to the bacterial fatty acid biosynthesis pathway and enable the biosynthesis of ladderane lipids. This possibility was explored in more detail in the present study, and its feasibility was compared to the alternative option, that the ladderane moieties of the lipids are derived from other biological intermediates by enzymes encoded in a separate, so far undiscovered gene cluster(s).

Comparative genomic analysis of the synteny of the *K. stuttgartiensis *FASII gene clusters led to the detection of very similar gene clusters in the unrelated, obligately anaerobic, cold adapted deltaproteobacterium *D. psychrophila*. This discovery was unexpected; if ladderane lipids were unique to anammox bacteria, we would not expect gene clusters for the biosynthesis of ladderane lipids in other bacteria. No ladderanes were detected experimentally in lipid extracts from cells of *D. psychrophila*, indicating that at least under the cultivation conditions applied, *D. psychrophila *did not produce ladderane lipids. However, *D. psychrophila *did produce unusual C_31:8 _and C_31:9 _hydrocarbons that have been previously identified in other psychrophillic bacteria and members of the *Planctomycetes *[19,20,49]. Non-isoprenoidal hydrocarbons are thought to be produced via decarboxylation of (e.g. C_32_) fatty acids [50]. The genome of *D. psychrophila *(and *K. stuttgartiensis*) did not encode other known pathways for PUFA biosynthesis, e.g. via polyketide synthesis.

The production of PUHC by *D. psychrophila*, the absence of known pathways of PUHC/PUFA biosynthesis in the *D. psychrophila *genome, the proposed role of PUHC as a precursor for ladderane biosynthesis in *K. stuttgartiensis *and the synteny between the FASII gene clusters, prompted us to re-analyse the extended gene clusters shared by these organisms. We found that it is possible that these clusters encoded a novel extension of FASII for the production of PUFAs.

To produce PUFAs from saturated fatty acids, double bonds have to be inserted by oxidative enzymes. There are oxidative enzymes encoded in the FASII gene clusters of *K. stuttgartiensis *and *D. psychrophila *that might perform the insertion of double bonds into a (growing) lipid chain. In *K. stuttgartiensis *there are two (Kuste3336, Kuste3607) and in *D. psychrophila *there are three (DP1860, DP2405, DP2406) open reading frames that encode for flavin containing amine-oxidoreductases (pfam01593, NCBI), similar in architecture to phytoene dehydrogenases. Phytoene dehydrogenases introduce double bonds into isoprenoid chains. However, it should be noted that isoprenoids are constructed from IPP units, already containing double bonds and that it is a more difficult task to introduce the first double bond into a saturated lipid. Therefore, it is probable that the phytoene dehydrogenase-like enzymes, encoded by *K. stuttgartiensis *and *D. psychrophila*, introduce subsequent double bonds into already unsaturated fatty acids. Another class of oxidative enzymes, the SAM radical enzymes are however more powerful, at the expense of adenosyl methionine they can perform a wide variety of reactions [26]. These enzymes could be involved in polyunsaturated lipid pre-or post-processing.

Pairwise comparison between the unusual gene products in *K. stuttgartiensis *and *D. psychrophila *(Figure 4b) indicates that many of the unusual *D. psychrophila *open reading frames are homologous to those in *K. stuttgartiensis *and therefore could drive a similar pathway. Remaining synteny between the *K. stuttgartiensis *and *D. psychrophila *FASII gene clusters could be related to the post-processing of the FASII products. The acyl-CoA ligase-like gene sequences encoded in both organisms (annotated as phenylacetyl-CoA ligase in *K. stuttgartiensis *and F_390 _synthetase in *D. psychrophila*) could perform the reaction for the first step of glycerol attachment and the unknown membrane protein (containing a putative lipid transport domain) may be involved in the transport and insertion of lipids into membranes, as hypothesised with similar Mmp gene sequences found in *Mycobacterium tuberculosis *[51].

**Figure 6:**
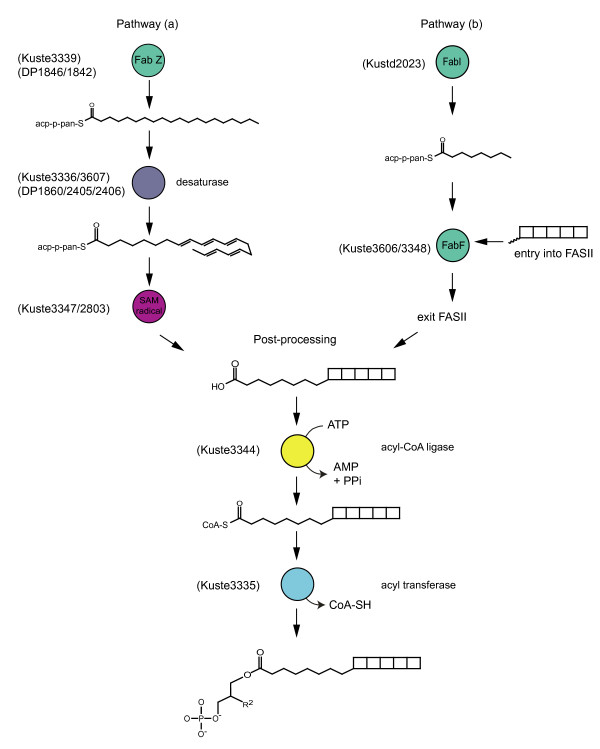
**Two hypothetical biosynthetic pathways for the formation of a C_20 _**[5]**-ladderane fatty acid**. Locus in *K. stuttgartiensis *(and *D. psychrophila*) identified as potentially responsible for catalysing steps in the pathway have been indicated. (a) the precursor PUFA molecule is synthesised from a C_20:0 _fatty acid using a desaturase to insert double bonds. The PUFA is then folded into a ladderane using a radical cascade mechanism, analogous to a previously proposed polycyclisation mechanism [48]. (b) Alternatively, the ladderane moiety could be synthesised via an as yet unknown pathway and brought into FASII via an unusual paralogue of FabF, attached to an acyl chain and released from FASII. The post-processing step is thought to be the same, irrespective of the pathway the ladderanes are synthesised. The last step shows the ester linkage of the ladderane fatty acyl group to L-glycrol 3-phosphate, during the synthesis of ladderane glycerophospholipids.

The similar isotopic signatures of *D. psychrophila *PUHC and monounsaturated fatty acids were consistent with the former being derived from the latter, with help of the gene products of the shared FASII gene clusters analysed above. However, no PUHC/PUFA could be detected in lipid extracts of *K. stuttgartiensis*. This experimental observation brings us to the essential, still remaining question: are PUHC/PUFA an intermediate of ladderane biosynthesis as previously proposed, or is PUHC/PUFA an independent product of the extended FASII pathway in *K. stuttgartiensis*? The failure to detect PUHC/PUFA is compatible with both scenarios. In the former scenario, PUHC/PUFA are intermediates (not end-products) of metabolism and could remain undetected. In the latter scenario, the cells may not have expressed PUHC/PUFA biosynthesis, because they were grown at 30°C and PUFA are typically produced in response to membrane adaptation to colder temperatures. The issue was further explored by detailed analysis of the encoded FASII gene products. If the first scenario would be true, the analysed gene clusters would encode the complete biosynthetic pathway for ladderanes. After all, the final cyclisation or cascade reaction necessary to produce ladderanes from PUFA could be performed by a single enzyme (Figure 6, pathway a). In the second scenario, we have to conclude that most of the biosynthetic potential of the gene clusters is dedicated to PUFA/PUHC biosynthesis and that ladderane biosynthesis in fact resides elsewhere on the genome. In this case, complete or partially completed ladderane moieties would have to be recruited into FASII (Figure 6, pathway b). In support of the cyclisation or radical cascading of PUFA into ladderanes scenario, our analyses showed that apart from the extensive synteny with some Deltaproteobacteria, the *K. stuttgartiensis *gene clusters also contained two genes with very limited sequence identity to genes found in other organisms. The following genes have the theoretical potential to be involved in cyclisations or oxidative cascades: Kuste2803 (a SAM radical enzyme with an additional B12 binding domain) and perhaps Kuste3347 (a SAM methyl transferase).

Comparative genomic analysis showed that multiple paralogues of *acp *and *fabF *are not unusual. They are known to be necessary for accommodating structural differences of the lipid backbone during chain elongation. However, the observed changes in key residues of the *fabF *active site in two divergent *fabF *paralogues of *K. stuttgartiensis *are quite rare (Table 2). In the reaction cycle of FabF, Cys163 first accepts the growing lipid chain from *acp*. Next, a second *acp *presents a malonyl group to the active site. His298 and His333 facilitate the decarboxylation of this malonyl group and so produce the acyl-carbanion necessary for the final step, the elongation of the growing chain with the acyl group. Mutagenesis studies have shown that the substitution of Cys163 to Ser163 (as in Kuste3348, the most divergent paralogue of FabF) still produces a functional phenotype but that the cystine at this position is important for the specificity of FabF for an *acp*-bound lipid chain [52]. His298 and His333 were both found to be indispensible for the decarboxylation of the malonyl-CoA. Interestingly, these two residues were completely substituted in all two previously mentioned paralogues of FabF. Therefore in conclusion, the active sites of these two enzymes are not compatible with the normal FabF chain elongation function. If they are functional and if their presence in the FASII gene clusters is not coincidental, these enzymes may recruit non-FASII products (for example, a ladderane moiety) into the FASII pathway. The analysis of alternative pathways for ladderane moieties (polyketide and isoprenoid biosynthesis) provided no additional clues on how these moieties may be synthesised.

## Conclusion

The present study has explored two propositions about biological ladderane biosynthesis: (1) Ladderane lipids are produced using polyunsaturated fatty acids (PUFA) as precursors; (2) The extended lipid biosynthesis gene clusters detected in the anammox bacterium *K. stuttgartiensis *encode a pathway for ladderane biosynthesis. Comparative genomics combined with experimental evidence has shown that the gene clusters of *K. stuttgartiensis *may encode a novel anaerobic pathway for PUFA biosynthesis that is also present in the unrelated organism *D. psychrophila*. The presence of an unusual SAM radical/B12 enzyme and SAM methyl transferase in *K. stuttgartiensis *is consistent with the idea that the oxidative cyclisation or radical cascading of PUHC or PUFA could produce ladderane lipids. However, the presence of multiple paralogues with radically different active site topology make it possible that ladderane moieties are synthesised via a presently unknown pathway and are recruited into fatty acid biosynthesis. Our study provides testable hypotheses for future experimental investigation.

## Abbreviations

PUFA: polyunsaturated fatty acid; PUHC: polyunsaturated hydrocarbons; SAM: S-adenosyl-methionine; GC/MS: gas chromatography/mass spectrometry; HPLC-MS/MS: high-performance liquid chromatography mass spectrometry/mass spectrometry; GC/CI/MS: gas chromatography chemical ionization mass spectrometry; EA-IRM/MS: elemental analysis isotope ratio monitoring mass spectrometry

## Competing interests

The authors declare that they have no competing interests.

## Authors' contributions

JER constructed phylogenetic trees and performed lipid extraction and analysis. MS performed the comparative genomic analysis. All authors contributed to interpreting the results and read, edited and approved the final version of the manuscript.

## Reviewers' comments

### Dr M. Galperin

Review of the manuscript by Rattray *et al. *"A comparative genomics study of genetic products potentially encoding ladderane lipid biosynthesis" submitted for publication in Biology Direct.

The paper by Rattray *et al. *addresses a very interesting aspect of lipid metabolism in the anammox bacterium *Kuenenia stuttgartiensis*, namely, the mechanism of formation of cyclobutane rings at the ends of the fatty acid residues of its phospholipids. Lipids with several linearly fused cyclobutane moieties have been shown to constitute a significant fraction of membrane phospholipids in *K. stuttgartiensis*, indicating the presence of a dedicated biosynthetic pathway catalyzing the formation of these unusual structures. This pathway can be expected to involve some novel biochemical reactions catalyzed by enzymes that remain to be characterized. In their earlier paper (ref. 25), the authors identified four operons encoding fatty acid biosynthesis in *K. stuttgartiensis *and analyzed the likely functions of the encoded proteins. They noted that the Kuste3352-Kuste3335 operon encodes two predicted S-adenosylmethionine radical enzymes and two SAM-dependent methyltransferases and suggested involvement of these enzymes in the ladderane lipid biosynthetic pathway.

In the current contribution, the authors have undertaken the first steps to verify this proposal. Using genome comparisons, they found a somewhat similar gene cluster in the genome of cultivable deltaproteobacterium *Desulfotalea psychrophila *and analyzed the lipid content of its membrane. They detected the presence in D. *psychrophila of *unusual polyunsaturated hydrocarbons, but not ladderanes. This observation suggested that gene products that are common for both organisms (including predicted radical SAM enzymes Kuste3343/DP1855 and Kuste3342/DP1853) are involved in synthesis of these polyunsaturated hydrocarbons, which could serve as precursors for ladderane formation.

I have mixed feelings about this manuscript. My major concern is that, while mechanisms of ladderane biosynthesis are indeed extremely intriguing, this manuscript provides only a minor contribution to the understanding of these mechanisms. Several specific comments are as follows.

1. The idea that SAM-related enzymes Kuste3347, Kuste3343, Kuste3342, and Kuste3338 are involved in ladderane biosynthesis has been published more than two years ago. The current paper would have looked far more convincing if there was an effort to characterize these enzymes experimentally by over expressing them in a suitable host and analyzing their properties *in vitro*. At the very least, this could have been done with their homologs from *D. psychrophila*.

#### Authors' response

We completely agree and would very much like to perform these types of experiments. However, the previous hypothesis "that they are involved in ladderane biosynthesis" provides little direction for future experiments. The present manuscript provides the clear hypotheses and directions needed for experimental testing. We believe that hypothesis generation is a significant aspect of biological research in the post-genomic era.

2. This paper reports the discovery in D. *psychrophila of *polyunsaturated hydrocarbons, but not ladderanes, but does not specify whether these polyunsaturated hydrocarbons contain macrocycles. If these polyunsaturated hydrocarbons are not cyclic, the presence in *D. psychrophila *of close homologs of Kuste3343 and Kuste3342 (DP1855 and DP1853, respectively) would strongly suggest that these enzymes are not involved in ring closure, and would therefore undermine the authors' key idea on the mechanism of ladderane formation. If these polyunsaturated hydrocarbons are indeed cyclic, there still remains the problem of finding suitable candidates to catalyze the carbon-carbon bonding that creates the cyclobutane rings.

#### Authors' response

The retention time and mass spectra clearly showed that the compounds in *D. psychrophila *were acyclic fatty acids with multiple double bonds. Fatty acids with large ring structures would have quite different mass spectra (e.g. contain an M-29) and retention time. We have amended the suggestion for the function of the similar radical enzymes in both organisms to playing a role in the pre- or post-synthesis of the polyunsaturated molecule. Possible candidates to catalyse cyclisations or oxidative cascades are Kuste2803 (a SAM radical enzyme with an additional B12 binding domain) and perhaps Kuste3347 (a SAM-methyl transferase).

3. A significant part of the manuscript is devoted to the phylogenetic analysis of the 7 paralogous members of the FabB/FabF family. The purpose of this analysis was not clear to me. How were the results expected to contribute to the ultimate goal of this work? There seems to be little doubt that all members of the FabB/FabF family are beta-ketoacyl synthases that participate in chain elongation. Further, what was the expected result from the analysis of the FabB/FabF active site residues? How does the finding that that "Kuste3348 is unique to *K. stuttgartiensis*" (apparently the main result of this analysis) help in understanding the mechanisms of ladderane formation?

#### Authors' response

In the new version of the manuscript this is illustrated in Figure 6. Our manuscript addresses two hypothetical pathways for ladderane biosynthesis. One of these depends on desaturation and ring closure, the other on the recruitment of a complete ladderane building block into FASII. The analysis of the FabBF paralogues addresses the latter possibility.

### Dr A. Osterman

In the study of Rattray et al., a comparative genomic approach was used to address an exciting (and still unsolved) mystery of ladderane biosynthesis in *Kuenenia stuttgartiensis*, a bacterium with environmentally and industrially important capability to anaerobically oxidize ammonia. By applying comparative genomic techniques, metabolic reconstruction, analysis of conserved chromosomal clusters, phylogenetic profiles and amino acid substitutions in paralogous enzymes, the authors identified candidate genes that may be responsible for the yet unknown biochemical transformations generating these fascinating polycyclical compounds. A compelling, albeit indirect, evidence is presented that ladderane formation may proceed via polyunsaturated fatty acid (PUFA) intermediates that undergo cascade cyclization by a SAM radical mechanism. This hypothesis, if proven correct, would indicate the existence of a novel variant of PUFA biosynthesis pathway, distinct from those previously described (eg in *Shewanella *spp). A conservation in the organization of extensive chromosomal clusters containing homologs of FAS-related genes between *K. stuttgartiensis *and a phylogenetically distant *Desulfotalea psychrophila *allowed the authors to hypothesize their involvement in related biosynthetic processes. Interestingly, the experimental analysis revealed the presence of PUFA but not ladderanes in the lipid composition of *Desulfotalea psychrophila*. A possible (although not the only one) interpretation of this finding is that both species share a PUFA synthesis pathway, whereas *K. stuttgartiensis *may have additional enzyme(s) converting PUFA intermediates to ladderanes. Candidate genes for such enzyme(s) were identified within the same chromosomal cluster. Although this study, taken alone, does not allow us to unambiguously discriminate between the two previously proposed models of ladderane biosynthesis, it sets the stage for the further direct assessment of its tentatively identified components.

### Dr J. Selengut

Prior to this work it had been established that 1) certain anammox bacteria biosynthesize ladderane lipids, 2) the genome of K. stuttgartensis contains larger fatty acid biosynthesis operons than is typical and 3) that these large operons contain SAM radical enyzmes. These observations are consistent with existing theories of ladderane genesis calling for the intermediacy of polyunsaturated fatty acids (PUFA).

The current work adds the following observations:

1) That *K. stuttgartiensis *contains the full complement of fatty acid biosynthesis genes, but additionally contains a number of homologs of the canonical FabB/F gene.

2) That these 'extra' homologs form a loose group of atypical FabB/F's with other sequences from R. baltica, a non-anammox planctomycete, and a number of deltaproteobacteria.

3) That some of these homologs from the two planctomycetes (and one of the deltas) do not contain many of the canonical conserved active site residues, strongly implicating a distinct function.

4) That two of the operons of *K. stuttgartiensis *containing these distinctive FabB/F homologs contains genes which are syntenic with similar operons in the deltaproteobacterium Desulfotalea psychrophila. The genes which are similar include the SAM radical enzymes and members of the amine oxidase family related to phytoene desaturases, both of which are reasonable to consider as possible actors in a fatty acid desaturation pathway.

5) That an alternative (known) pathways for PUFA biosynthesis via polyketides was absent from both *K. stuttgartiensis *and *D. psychrophila*.

6) That, although ladderane production could not be established in *D. psychrophila*, polyunsaturated hydrocarbons (PUHC) could be observed prominantly in that organism, and that natural isotope patterns of these PUHC's are consistent with their derivation from FA's.

The authors do not make any substantial conclusions based on these observations, and indeed, I concur that none are warranted. The connection between these operons in *K. stuttgartiensis *and the production of ladderane lipids is based on reasonable hypotheses, the absence of obviously better candidates and circumstantial evidence. Until such time as one can isolate and manipulate a ladderane-producing bacterium or produce and study the gene products thereof, this must be the case.

The possibility exists and must be respected that the observed operons have a purpose other than the production of ladderanes or their precursors. The chain of inference that goes from ladderane production in *K. stuttgartiensis *to non-standard fatty acid biosynthesis operons in *K. stuttgartiensis *to syntenic operons in *D. psychrophila*. to PUHC production in *D. psychrophila *is sufficiently weak at each step that little confidence is gained that the proposed mechanism of ladderane biosynthesis is correct. This uncertainty is properly noted by the tone of the final paragraphs of this manuscript.

Nevertheless, considering the difficulties involved in obtaining anammox bacteria in a form conducive to laboratory studies, the current work points to a number of interesting candidate proteins for study by expression and isolation from heterologous hosts.
